# Toward quantitative understanding on microbial community structure and functioning: a modeling-centered approach using degradation of marine oil spills as example

**DOI:** 10.3389/fmicb.2014.00125

**Published:** 2014-03-26

**Authors:** Wilfred F. M. Röling, Peter M. van Bodegom

**Affiliations:** ^1^Molecular Cell Physiology, Faculty of Earth and Life Sciences, VU University AmsterdamAmsterdam, Netherlands; ^2^Systems Ecology, Department of Ecological Sciences, Faculty of Earth and Life Sciences, VU University AmsterdamAmsterdam, Netherlands

**Keywords:** systems biology, flux balance analysis, metagenomics, bottom-up modeling, microbial communities, marine oil spills

## Abstract

Molecular ecology approaches are rapidly advancing our insights into the microorganisms involved in the degradation of marine oil spills and their metabolic potentials. Yet, many questions remain open: how do oil-degrading microbial communities assemble in terms of functional diversity, species abundances and organization and what are the drivers? How do the functional properties of microorganisms scale to processes at the ecosystem level? How does mass flow among species, and which factors and species control and regulate fluxes, stability and other ecosystem functions? Can generic rules on oil-degradation be derived, and what drivers underlie these rules? How can we engineer oil-degrading microbial communities such that toxic polycyclic aromatic hydrocarbons are degraded faster? These types of questions apply to the field of microbial ecology in general. We outline how recent advances in single-species systems biology might be extended to help answer these questions. We argue that bottom-up mechanistic modeling allows deciphering the respective roles and interactions among microorganisms. In particular constraint-based, metagenome-derived community-scale flux balance analysis appears suited for this goal as it allows calculating degradation-related fluxes based on physiological constraints and growth strategies, without needing detailed kinetic information. We subsequently discuss what is required to make these approaches successful, and identify a need to better understand microbial physiology in order to advance microbial ecology. We advocate the development of databases containing microbial physiological data. Answering the posed questions is far from trivial. Oil-degrading communities are, however, an attractive setting to start testing systems biology-derived models and hypotheses as they are relatively simple in diversity and key activities, with several key players being isolated and a high availability of experimental data and approaches.

## Introduction: key questions in microbial ecology and oil spill bioremediation

Microbes are prime catalysts of environmentally and societally important ecosystem processes, such as the biodegradation of spilled oil. Yet, the large complexity of microbial communities and technical limitations have long prevented the accurate description of microbial communities, let alone establishing the contribution of microorganisms to ecosystem functioning (Fuhrman, 2009). Metagenomics and related microbial ecological approaches are nowadays employed, aiming to answer major questions in microbial ecology:

1. How do microbial communities assemble in terms of functional diversity, species abundances and organization, and what are the drivers of community assembly?
2. How do the functional properties of microorganisms scale to processes at the ecosystem level?
3. How does mass flow between species, and which factors and species control and regulate fluxes, stability and other ecosystem functions?
4. Can generic rules be derived in microbial ecology, what drivers underlie these rules, and do these rules resemble rules in plant and animal ecology?
5. What information is needed for predicting and engineering microbial communities and their functioning?

However, while metagenomic approaches lead to large data sets, the cataloguing of genes itself provides limited insight, and may lead over time to disappointment in microbial ecology and its practitioners (Prosser, 2013). The application of theory from other research areas is needed to provide structure, mechanistic insight and, ultimately, predictive power (Prosser et al., 2007; Raes and Bork, 2008). In this paper, and in contrast to many reviews on individual approaches, we argue for a novel, bottom-up mathematical framework that combines several existing approaches to better understand microbial communities and their activities. We subsequently indicate what is required to make such framework successful, and identify a need to link microbial physiology to quantitative concepts in order to advance microbial ecology via modeling-based approaches.

**Figure 1:**
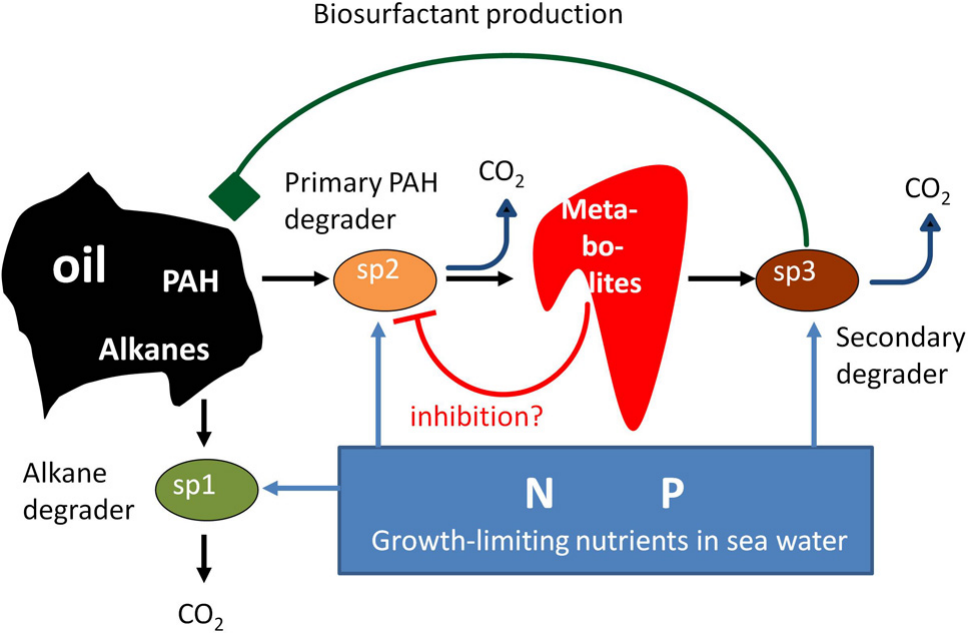
**Several potential interactions during oil-mineralization that may affect the rate and extent of biodegradation of marine oil spills**. Alkane- and PAH-degrading specialists compete for limiting nutrients in seawater. We extrapolate findings on a benzo[a]pyrene degrading, soil-derived consortium (Kanaly et al., 2002) to marine oil spills (Head et al., 2006) to indicate potential positive mutualistic interactions. The consortium was found to contain a key member (indicated by Sp3) that was unable to degrade the PAH benzo[a]pyrene but excreted factors that aided its degradation and presumably grew on metabolites excreted by other, benzo[a]pyrene-degrading community members (indicated by Sp2).

We are aware that the job ahead is tremendous and far from trivial. The biodegradation of marine oil spills provides a suitable and realistic starting point to achieve our goals, and also to pose and test specific hypotheses. Many molecular microbial ecology-centered studies have appeared, especially motivated by the 2010 Deepwater Horizon oil spill in the Gulf of Mexico and a desire to know what happened to its microbial communities and their degrading activities (e.g., Camilli et al., 2010; Hazen et al., 2010; Lu et al., 2012; Mason et al., 2012). These studies have provided insights on the major contributing species and their interactions (Head et al., 2006). Upon a spill, microbial biodiversity is strongly reduced, after which oil components are sequentially degraded (Head et al., 2006). First, growth of alkane-degrading specialists occurs, with *Alcanivorax* species contributing up to 90% of cell counts. Next, polycyclic aromatic hydrocarbon (PAH) degrading microorganisms, like *Cycloclasticus*, take over. Conceptual models exist on the importance of nitrogen and phosphorus in oil-biodegradation and on the interactions between functional groups of microorganisms (e.g., Head et al., 2006; Figure 1). Several important aspects of the biodegradation of spilled oil are still not well understood: why do certain specialists become dominant during oil-degradation, why are oil compounds sequentially degraded with first the relatively harmless alkanes being removed before the more toxic PAHs are attacked? We hypothesize that multispecies metabolic network-based modeling approaches, as outlined in the next sections, will be able to provide the answers. The obtained insights may subsequently contribute to the design of more effective oil spill bioremediation approaches, and enable the faster removal of toxic PAHs.

## Why model, and how to model?

Biology is predominantly non-linear in character, for instance consider the biology text-book examples of Michaelis-Menten enzyme kinetics and Lotka-Volterra prey-predator interactions. The non-linearity in combination with the immense complexity of microbial communities makes it empirically extremely difficult to decipher the respective roles of each player in the provision of community-derived fluxes and community functioning in general, in dynamic environments with varying chemical and physical conditions. These aspects make it obvious that mathematical approaches are needed to ever come close to understanding microbial communities and functioning, and tackle key questions in microbial ecology. Introducing ecological theory has been suggested as one avenue to advance microbial ecology (Prosser et al., 2007), and for instance resource-ratio theory has been applied to oil spill bioremediation (Röling et al., 2002). While extending ecological theory into microbial ecology is undoubtedly very important, a key difference between plants and animals on the one hand and micro-organisms on the other hand is the enormous physiological and biochemical diversity in microorganisms. Thus, we propose to model within a microbial eco-systems biology context by extending and integrating current systems biology (explained in more detail from Section “What is Needed to Mechanistically Model Complex Communities: The Big Lines” onwards). Systems biology has considerably enhanced insight into the functioning of individual microbial species, and the employed approaches may be adapted and applied to microbial ecology to contribute to improved understanding of microbial community composition and functioning (Röling et al., 2010; Zengler and Palsson, 2012).

Systems biology comprises an iterative cycle of experimentation, data analysis and modeling, hypothesis formulation and testing. Bottom-up systems biology approaches have led to large insights in the functioning of single species by examining the mechanisms through which functional system properties arise in the interactions of components in the system. These approaches require measures on physicochemical and kinetic properties of the components to model system properties (Bruggeman and Westerhoff, 2007). Bottom-up systems biology approaches can direct medicine development (Bakker et al., 2002) and metabolic engineering of microbial strains applied in biotechnological processes (Hoefnagel et al., 2002; Izallalen et al., 2008). We envision that in a similar fashion we will be able to design environmental “medicines”, e.g., the application of process-specific inhibitors, biostimulation with limiting nutrients or bioaugmentation to resolve missing or suboptimal microbial functions.

**Figure 2:**
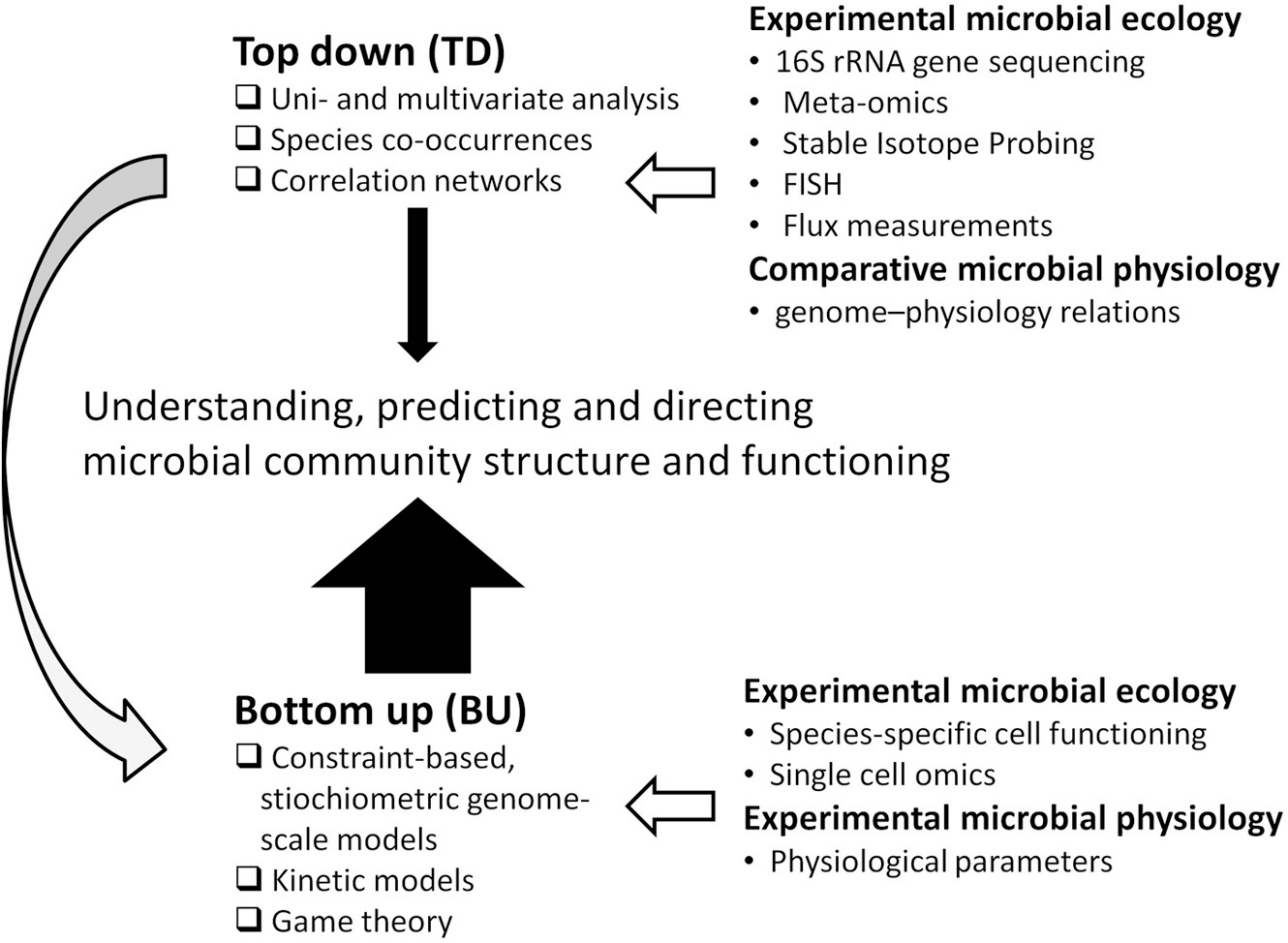
**Bottom-up (BU) vs. top–down (TD) approaches to model microbial community structure and its functioning**. In this paper we advocate an approach that is strongly BU (as indicated by the thick, solid arrow) with some TD modeling, also to aid the BU modeling (as indicated by the gray arrow). The relation of BU and TD approaches to experimental microbial ecology and physiology is indicated by open arrows.

This contrasts to top–down systems biology approaches, identifying molecular interaction networks on the basis of correlated molecular behavior derived from (meta)genome-wide “omics” studies (Bruggeman and Westerhoff, 2007). The popularity of these approaches coincides with a generally increasing popularity of multivariate statistical approaches in microbial ecology (Figure 2; Raes and Bork, 2008). Indeed, they bring the field forward and will do so for the coming time as many microbial systems are still poorly characterized. Yet, such models are phenomenological, and have limited predictive value, while frequently employing relations between properties that are assumed to be linear, even though biology is generally non-linear.

Bottom-up approaches may provide the mechanistic insights required to truly advance microbial ecology in the future. Bottom–up approaches, however, can be parameter-rich and are sensitive to undetermined factors, which are already important drawbacks for modeling single species (Bruggeman and Westerhoff, 2007). These problems are further amplified for complex microbial communities. Thus, there is a need to integrate bottom-up models with top–down approaches for better completeness, and reduce the complexity of the models (Figure 2).

## What is needed to mechanistically model complex communities: the big lines

The abovementioned complexity of microbial communities provides considerable challenges for bottom-up modeling. Clearly, an “abstraction” of reality is needed to understand community structure and functioning. However, a balance (that is, modeling approaches not being too detailed, but also not too simple) is needed to prevent arriving at a dead end, far from the goal of answering major questions in microbial ecology.

A good modeling practice is to start with a relatively simple model, describing a relatively simple community, and then stepwise increase complexity. Modeling and experiments (see Box 1“Experimental approaches in microbial ecosystems biology”) would need to go hand in hand. Unstructured (that is, well-mixed) simple “communities” consisting of a few interacting species, in a system that is closed (in the sense that no other species can invade), provide an initial starting point. Later, structure and higher diversity can be introduced, and subsequently dynamics in community structure, by allowing species immigration both in modeling and in experimentation. Biodegradation of marine oil spill provides an excellent starting point for such practice, since often a limited number of microorganisms with specific functions appear to play key roles (e.g., Head et al., 2006; Figure 1). Important representatives of these functional groups have been cultured (Dyksterhouse et al., 1995; Yakimov et al., 1998), allowing for controlled experiments. Furthermore, laboratory experimental designs mimicking beach oil spills have already shown their utility to test hypotheses on oil spill bioremediation (Röling et al., 2002).

Box 1 **Experimental approaches in microbial ecosystems biology.**

Experiments will be needed to parameterize community models but also to test model-derived hypotheses (Figure 2). What one in particular would like to measure is: what activity does a certain species perform in a community, and how fast? With whom does it interact? The current experimental tool box is already quite complete to answer these questions and to reveal how interactions with other species affect a species' metabolism.

Species-specific activities can be quantified by combing Fluorescent *In Situ* Hybridization with the uptake of stable (Musat et al., 2008; Finzi-Hart et al., 2009) or radioactive isotope labeled (Nielsen et al., 2003) or fluorescent substrates (Muller and Nebe-Von-Caron, 2010), or by probe-based capturing of labeled DNA or RNA (Van Mooy et al., 2004; Van Mooy and Devol, 2008). Combinatorial fluorescent labeling and spectral imaging (Valm et al., 2011) can resolve up to 15 phylogenetic target groups at one time by FISH. This approach combined with high throughput flow cytometry with post-sorting analysis hold great promise for the future (Pel et al., 2004; Muller and Nebe-Von-Caron, 2010). Also antibodies can be used to separate species from microbial consortia and to determine species-specific characteristics (Pelz et al., 1999).

Isotopically labeled substrates also enable tracking substrate flow within and between cells. Metabolic flux analysis is the experimental counterpart of FBA to measure realized internal fluxes on basis of measuring external fluxes, mass balancing and reaction stoichiometry. Due to the occurrence of, e.g., cycles or multiple pathways, ^13^C Metabolic flux analysis is needed to resolve internal fluxes (Sauer, 2006). Generally, the positional isotope distribution of ^13^C in specific amino acids incorporated in cellular protein is determined and the distribution of these so-called isotopomers are fitted to a mathematical model of central metabolism that tracks the flow from ^13^C labeled substrate to amino acids to obtain the flux distribution of the species under study. Low internal concentrations currently hamper metabolite-based ^13^C flux analysis (Sauer, 2006). In contrast, extracellular products can occur in high concentrations, their isotopomeric analysis enable determining the fluxes through major pathways in human intestinal fermentation of glucose (De Graaf et al., 2010).

Combined with stable isotope probing (SIP), isotopomeric analysis provides information on cross-feeding of metabolites between species (Kovatcheva-Datchary et al., 2009). SIP can also provide information on interactions by predation (Lueders et al., 2006). In SIP an stable isotope-labeled compound is added to an environmental sample and the isotope-labeled biomarkers that are produced in the target organisms are analysed at the community scale (e.g., Pilloni et al., 2011). SIP addressing DNA, rRNA and mRNA provide complementary information on the growth and activity of microorganisms. DNA-based SIP will measure primarily newly formed cells, while RNA-based SIP will address also non-growing microorganisms and is highly dynamic, especially mRNA (Dumont et al., 2011). Jehmlich et al. (Jehmlich et al., 2009) developed a concept for analyzing carbon and nitrogen fluxes in microbial communities by employing protein-based SIP in metabolic labeling experiments with stable isotope labeled substrates.

The relatively simple modeling approaches should, however, allow for mechanistic insights. For instance, Taffs et al. (2009) constructed a metabolic network to describe community activity by considering the community as a single meta-organism. They connected genes via their inferred metabolites without specifically taking into account that connected genes may have belonged to different species (Taffs et al., 2009). Such boundary-free approaches appear attractive in the current metagenomics era, as generally the exact relationship between a gene and the cell that contained it, is lost in metagenomics. However, when comparing such cell boundary-free models with models that described individual species in a consortium, clear differences were revealed. Boundary-free models suffered from the likelihood of including infeasible reactions and the inability to obtain biomass estimates for individual species (Taffs et al., 2009). Also by simple reasoning one must conclude that some degree of compartmentalization is needed in modeling microbial communities: microbial activities depend on enzyme activities, thus on Michealis-Menten kinetics that relate metabolite concentrations to activities. The intracellular and extracellular concentration of a particular exchangeable metabolite is usually different, suggesting that top–down approaches have limited utility and cell boundaries must be included instead.

“Rules” on microbial cell and community functioning may indicate how one could compartmentalize a complex community and in which detail these compartments should be described. Compartmentalization can be at the level of the individual cell, strain, species, or at a higher level (e.g., functional group). A higher level is preferable, as it minimizes the number of compartments in the model. The observation that a number of *Shewanella* species and *Escherichia coli* were highly similar in growth characteristics (growth rate, fermentation products) and in intracellular fluxes through their major metabolic pathways, led Tang et al. (2009) to introduce the concept of the metabotype. Species with similar metabolic phenotypes are grouped into a metabotype, irrespective of possible differences in their phylogeny. This concept may pave the way to model microbial community functioning on basis of a limited number of compartments describing the different functional types. Spilled oil is degraded by marine microorganisms that are generally specialized in the degradation of either alkanes or PAHs independent of phylogeny (Head et al., 2006), and which may possibly be divided along these lines in metabotypes (Figure 1).

The detail in which each compartment is described, must be considered too. Microorganisms respond to their biotic and abiotic environment, and changes therein, by adapting their biochemistry and physiology. Thus, models should enable simulation of this kinetic flexibility of microorganisms. An empirical Monod equation describing the dependence of growth rate on a single limiting substrate, combined with a fixed-value growth yield and maintenance energy, is frequently used to model microbial growth, but is inadequate for our purpose of understanding growth and activity in environmental settings that are complex and dynamic, such as oil-polluted marine environments. Oil consists of thousands of compounds belonging to a few major classes, such as alkanes and PAHs (Head et al., 2006). While oil is degraded by specialists (Figure 1), these specialists can often degrade a range of molecules belonging to a specific class (Dyksterhouse et al., 1995; Yakimov et al., 1998; Schneiker et al., 2006). In addition, oil-degradation is strongly affected by nitrogen and phosphorus limitation (Head et al., 2006; Figure 1). Monod equations have limited ability to describe multiple substrate use or changes in type of growth limitation (Kovarova-Kovar and Egli, 1998). Growth yields and maintenance energy requirement are not constant within a single species, but depend on growth conditions (e.g., Van Verseveld et al., 1984; Van Bodegom, 2007).

The other extreme is to consider the complete genetic and metabolic make-up of a species, which seems infeasible since a microbial genome contains thousands of genes, encoding thousands of proteins that can act on thousands of metabolites. For most enzymes, quantitative information, like affinity constants and maximum activities, is limited, and measuring all these parameters is cumbersome, if even feasible. Many genes and pathways appear to be only expressed and active under a few conditions, or are largely invariant to changes in environmental conditions (e.g., Daran-Lapujade et al., 2007; Tang et al., 2007; Kelk et al., 2012). This suggests that it is possible to reduce compartment complexity in order to determine genome-based metabolic fluxes with a minimal use of kinetic parameters.

Flux-analysis approaches as initially developed in cellular systems biology, like Metabolic Control Analysis (MCA; Kacser et al., 1995) and Hierarchical Regulation Analysis (HRA; Ter Kuile and Westerhoff, 2001; Daran-Lapujade et al., 2007), can contribute to establishing the detail in which a particular compartment needs to be described. MCA and HRA quantify and identify the importance of individual cellular components (e.g., enzymes) and processes (e.g., transcription) for systems-level metabolic fluxes and have aided in establishing how biochemical systems change upon perturbation. These approaches have been extended to analyse metabolic and trophic interactions among species and between species and their abiotic environment (Allison et al., 1993; Getz et al., 2003; Röling, 2007; Röling et al., 2007). For instance, MCA is an advanced sensitivity analysis framework that reveals, computationally or experimentally, how modulation of enzyme activities affects metabolic fluxes and metabolite concentrations in a cell (Kacser et al., 1995). It generates so-called control coefficients that quantitatively indicate the importance of an enzyme for a given process. Control analysis showed that at the cellular level, flux control is often mostly with the transporter (Bakker et al., 1999), and that in anaerobic, organic matter degrading communities, flux control is dominantly with the primary fermenting microorganisms (Röling et al., 2007). These observations suggest that in describing the compartments one should especially focus on uptake kinetics, while in anaerobic organic matter degrading networks the compartment(s) representing primary fermenting microorganisms needs most detail. Also in oil spill degradation several physiological groups of species interact, and the rate of oil-degradation might, for instance, be controlled considerably by biosurfactant-producing community members that live on metabolites excreted by oil component-degrading microorganisms (Figure 1).

## Flux balance analysis and microbial ecology

**Figure 3:**
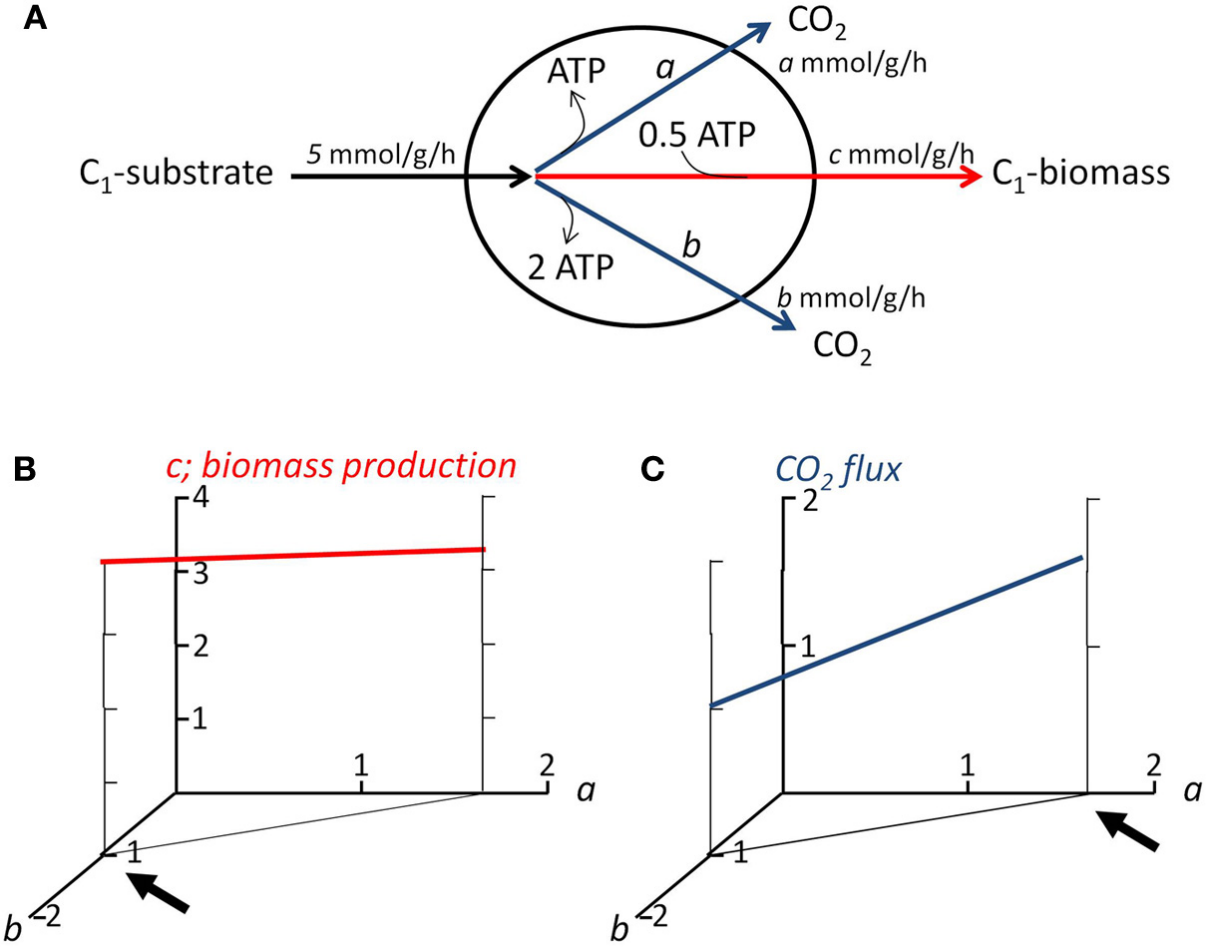
**Demonstration of flux balance analysis and objective functions**. **(A)** Simplified representation of the metabolism of a microorganism, e.g, an alkane-degrader. The organism is assumed to consume substrate (formulated as C_1_-unit) at a rate of 5 mmol per gram biomass per hour. It grows, at flux *c* (in mmol per gram biomass per hour) by producing offspring from the substrate, which costs 0.5 ATP per C_1_-unit biomass produced. The ATP needed for anabolism can be derived from two catabolic pathways, pathway *a* produces 1 ATP per mole C_1_-substrate consumed, pathway *b* 2 ATP. Two flux balances containing three unknowns (*a*, *b*, and *c*) apply in steady state: 1. the carbon going into the cell, must come out: *a* + *b* + *c* = 5; 2. there can be no net production or consumption of ATP: *a* + 2*b*− 0.5*c* = 0. As a result, no unique solution is obtained for fluxes *a*, *b*, and *c*, an infinite number of solutions lay along the line 3*a* + 5*b* = 5. **(B)** Relationship between the fluxes through the two catabolic pathways, and associated biomass production. If the objective function of the microorganism is to maximize biomass production, than it should use only pathway *b* for ATP production (indicated by arrow); **(C)** Relationship between the fluxes through the two catabolic pathways, and associated CO_2_ production. If the objective function of the microorganism would be to maximize CO_2_ production, than it should use only pathway *a* (indicated by arrow). Thus, the objective functions allow to obtain an unique solution.

Flux balance analysis (FBA) is currently the systems biology approach that appears most suited for the tasks outlined above. FBA is the stoichiometric analysis of a genome-derived metabolic network and allows calculating the possible metabolic flux distributions and other system properties (e.g., biomass yield, growth rate, ability to consume or produce certain chemicals), without requiring detailed kinetic information (Feist et al., 2009; Figure 3). To describe which fluxes are possible in a particular condition, physiological constraints (e.g., substrate uptake rates, biomass composition; Section “Deriving Microbial Physiological Parameters for Application in FBA”) and optimization principles (Section “Optimization Principles for Multispecies FBA: Microbial Growth Strategies”; Figure 3) are applied. Even when the metabolic network is solely based on gene presence, FBA can provide accurate predictions of system properties of a single species (Feist et al., 2009).

In recent years, FBA approaches have been developed that further aid in more accurately describing the physiology of individual species. These approaches utilize experimental data on gene expression (Covert et al., 2004; Becker and Palsson, 2008; Shlomi et al., 2008) or implemented theoretical considerations informed by principles from biochemistry and genomics (e.g., Beg et al., 2007; Henry et al., 2007; Chandrasekaran and Price, 2010). Generating metabolic network models, and in particular fine-tuning these models, used to be time consuming but is now considerably aided by high-throughput, internet-based resources (Henry et al., 2010; Boele et al., 2012).

While FBA was initially developed for the analysis of fluxes under steady state conditions, Mahadevan et al. (2002) extended it to dynamic environments, by inserting an FBA model into an ODE (operational differential equation) model. By considering the cell to be in pseudo-steady state at each time point, growth rate and growth yield were calculated at each time point (Mahadevan et al., 2002). Nutrient uptake was described with Michaelis-Menten kinetics to generate dynamics. This dynamic FBA (dFBA) is especially of interest to ecology, since ecosystems are generally dynamic over time and space. dFBA is particularly relevant for marine oil spill biodegradation, since for instance fast growth of *Alcanivorax* occurs within a few days after a spill (Head et al., 2006). dFBA models of microorganisms have been successfully introduced into reactive transport models (Scheibe et al., 2009; Zhuang et al., 2011), which are used in hydrology to describe biogeochemical processes and physical transport processes in detail. Also to describe and understand the growth of key microorganisms on oil spilled on beaches, reactive transport models integrating the supply and removal of nutrients and cells by tidal cycles would be beneficial.

Increasingly, FBA is also applied to describe ecological interactions in simple consortia consisting of two to three species, providing a basis for studying more complex communities (Section “Constructing Metabolite-Based Microbial Interaction Networks”). The first multispecies flux balance analysis was conducted by Stolyar et al. (2007). Reduced metabolic network descriptions of fermenting, hydrogen-producing *Desulfovibrio vulgaris* and hydrogenotrophic *Methanococcus maripaludies* were combined to describe their mutualistic interactions, with the medium as a third compartment through which the species interacted. Several ecologically relevant characteristics, such as flux of metabolites and ratio of the two species, were predicted accurately. It revealed that interspecies transfer of hydrogen was essential in the interaction, while format was not. Competition, and the resulting species ratios, were accurately modeled for iron-reducing *Rhodoferax* and *Geobacter* along the groundwater flow path through an uranium-polluted aquifer, by including simple Michaelis-Menten kinetics to describe nutrient uptake rates that acted as constrains in a dynamic FBA model (Zhuang et al., 2011). The type of limiting nutrient (carbon or nitrogen) determined which species won the competition. Nutrient limitation plays a key role in the degradation of marine oil spills, as do competitive and mutualistic interactions between alkane- and PAH-degraders (Head et al., 2006; Figure 1). We hypothesize that by comparing the growth of oil-degrading marine microorganisms, using multispecies, metabolic network-based based models that are integrated into reactive transport models in order to take into account nutrient availability and other relevant environmental characteristics, we will be able to decipher why *Alcanivorax* generally dominates and outcompetes other oil-degraders.

While the genomes of several *Alcanivorax* species and other marine microorganisms capable of alkane- or PAH-degradation have been sequenced (Brooijmans et al., 2009), for none of them has an FBA model on their oil-degradation yet been reported. The metabolic network of the PAH-degrading *Mycobacterium vanbaalenii* PYR-1 has a funnel-like topology, in which many peripheral pathways, acting on a wide range of PAHs differing in complexity, converge to a widely conserved central pathway (Kweon et al., 2011). This organization may enhance input diversity with the controlled production of limited outputs, allow for more coordinated regulation, and ensure more efficient metabolic flow with reduced metabolite dissipation (Kweon et al., 2011). The metabolic networks of other marine microorganisms that degrade alkanes or PAHs likely have a similar funnel-like topology (e.g., Schneiker et al., 2006). We expect that FBA models that also consider the costs of protein synthesis, will reveal that diauxic growth by first depleting the smallest and least complex oil compounds enables the fastest growth of an oil-degrader. A consequence might be that after an oil spill the growth of microorganisms that are capable of using a small range of relatively simple alkanes or PAHs is favored over the growth of microorganisms that degrade a wider range that also include the more complex oil compounds, as the latter have to bear the costs for the genes and proteins required for these activities. The subsequent growth of microorganisms capable of the removal of the more complex and more toxic oil components could then be hampered by the low availability of phosphorous and nitrogen, as most of these nutrients will be contained in the biomass of the pioneering species. Community-level, multispecies FBA will be useful to understand such microbial interactions during oil-degradation and to design new bioremediation strategies.

Community-level FBA reveals fluxes through species (analogous to the fluxes through enzymes within a cell in single species FBA). Conventional multispecies FBA does not provide cell numbers of individual species (again analogous to single species FBA where also the enzyme concentrations mediating the fluxes are not considered), while this is of major interest to microbial ecologists to understand and predict community structure. Recently, a few multispecies FBA approaches have been published that allow for predicting the cell numbers of individual species, for a wide range of microbial interactions (Zomorrodi and Maranas, 2012; Khandelwal et al., 2013).

## Constructing metabolite-based microbial interaction networks

So far, multispecies FBA modeling has been applied for up to three species (Taffs et al., 2009) and, unfortunately, no examples are available for oil-degradation yet. This number of interacting species is still limited, certainly in light of the thousands of species that can occur in just one gram of soil, thus providing a formidable challenge to FBA. High throughput sequencing of phylogenetic marker genes, in particular 16S rRNA genes, nowadays allows to establish relations between species and to construct microbial networks based on correlations between marker-derived species abundances. However, species interrelationships do not inform directly on the nature of these interactions (Faust and Raes, 2012), let alone the type of metabolites involved.

Recently, Langille et al. (2013) described a computational approach to predict the functional composition of a metagenome using 16S rRNA marker gene sequences, under the assumption that phylogeny and function are sufficiently linked. This approach to predict genome content on basis of a 16S rRNA sequence could be integrated into 16S rRNA-gene based microbial interaction networks to predict how the species may metabolically interact. The construction of metabolic networks from experimentally or computationally derived (meta)genome data for FBA itself provides information on the potential interactions between species: the metabolites taken up and produced by a species' network can be predicted (Borenstein et al., 2008; Handorf et al., 2008), allowing for the construction of metabolite-based microbial interaction networks (Borenstein and Feldman, 2009; Röling et al., 2010). Likewise, metagenomics can contribute information on prey-predator interactions, which is challenging in multispecies FBA as predation relates to prey size and prey aggregation behavior (Matz and Kjelleberg, 2005), properties that do not appear from a metabolic network. However, single cell genomics on marine protists revealed which preys they had ingested, and also indicated phage-cell interactions (Yoon et al., 2011). Both protists and phages may affect oil spill biodegradation (Röling et al., 2002; Head et al., 2006).

Also systematic literature mining approaches can enhance our understanding on the organization of microbial interaction networks (Chaffron et al., 2010; Freilich et al., 2010). For instance, a network constructed based on published bacteria co-occurrences showed that this network clustered into species-groups that showed relations between resource competition, metabolic yield and growth rate that correspond to the r/K selection theory (Freilich et al., 2010).

Similar approaches as described above might be applied to marine oil spill degrading microbial communities, by utilizing the many descriptive and empirical studies that have appeared in recent years, especially after the 2010 Deepwater Horizon oil spill (e.g., Camilli et al., 2010; Hazen et al., 2010; Lu et al., 2012; Mason et al., 2012). By combining their data, species-species interactions during different phases of oil spill biodegradation may be inferred to subsequently construct metabolite-based microbial interaction networks which can subsequently be analyzed by multispecies FBA approaches as described in the previous section. By focusing first on key oil-degraders such as *Alcanivorax* and *Cycloclasticus* and their interaction partners (Figure 1), we hypothesize that we can identify species that are either synergetic or antagonistic to degradation of specific classes of oil components and also pinpoint the mechanisms (e.g., metabolic network characteristics) behind these interactions. These species may subsequently be stimulated or inhibited to favor the degradation of relatively more toxic PAHs over alkanes.

## Optimization principles for multispecies FBA: microbial growth strategies

FBA models are in general underdetermined: the number of variables in the equations to solve is larger than the number of equations themselves, even when constraints such as maximum uptake rates of nutrients and biomass composition are included (Figure 3A). Therefore, FBA uses optimization criteria, or objective functions, to describe a species' physiology. Optimization principle(s) are based on the assumed or determined growth strategies of the organism under study, such as maximization of biomass production. Figures 3B,C demonstrate two different objective functions and their impact on flux distribution over anabolic and catabolic pathways. Often, it is assumed that cells aim to maximize their growth yield (Figure 3B), however this strategy is by no means an universal principle (Schuetz et al., 2007; Schuster et al., 2008). Even a single species can employ different strategies depending on the prevalent growth conditions, such as nutrient scarcity or excess (Schuetz et al., 2007). On basis of ^13^C flux analysis of nine bacteria, metabolism was shown to operate close to the so-called Pareto optimal surface of a three-dimensional space defined by competing objectives (biomass yield, ATP yield, minimum sum of absolute fluxes) (Schuetz et al., 2012). Flux states were proposed to evolve under the trade-off of two principles: optimality under one given condition and minimal adjustment between conditions (Schuetz et al., 2012).

The growth strategies of oil-degrading microorganisms, and their dependence on environmental conditions and species identity, are not known. However, we envision that knowledge on growth strategies will be key to understanding and directing the degradation of spilled oil and associated community dynamics. If for instance alkane-degrading microorganisms aim to maximize their biomass production (Figure 3B), a consequence might be that most of the often growth-limiting nitrogen and phosphorus will end up in their biomass (Figure 1). Hence, after alkanes are depleted, little nutrient will be available to enable substantial biomass production of PAH-degrading microorganisms and fast degradation of PAH. If on the other hand alkane-degrading microorganisms aim to maximize CO_2_ production from alkanes, for instance as a strategy to avoid that competitors can use these alkanes, less biomass will be formed per molecule alkane degraded (Figure 3C) and more nutrients will be available for growth of PAH-degraders (Figure 1). We expect that by comparing experimental growth in mono- and mixed cultures to FBA models employing different optimization criteria the growth strategies of oil degrading microorganisms will be revealed.

For what cells are optimized in complex, multispecies environments is in fact also not well known. Probabilistic cellular decisions on costs and benefits may be taken at three levels in such environments (Perkins and Swain, 2009): cells firstly have to derive from noisy signals the current and potential future states of their extracellular environment. Second, given those anticipated future states, microbes must weigh the costs and benefits (in terms of fitness and its optimization) of each potential response, at the level of the individual. Finally, the cells must decide in the presence of other (potentially competing or cooperating) decision makers, at the level of the population and the community. Competition between cells and species in communities may force strategies that appear suboptimal: a strategy with lower fitness in environments without competition, might be successful in environments with competition, an outcome well known from plant ecology too (e.g., Anten and During, 2011; Falster et al., 2012).

Game theory, which studies strategic decision making, can provide a better approach than conventional optimization to study the dynamics and outcome of the development of microbial communities, by capturing evolutionary considerations as affected by interactions between microorganisms (Pfeiffer and Schuster, 2005). The trade-off of growth yield vs. growth rate is an example of such dilemma (Pfeiffer et al., 2001; Kreft and Bonhoeffer, 2005). This trade-off is based on irreversible thermodynamics. Chemotrophic organisms obtain their energy by the degradation of substrates into products with lower free energy. The free energy difference between substrate and product is used for two purposes: ATP production for biomass growth and the thermodynamic driving force of the degradation reaction. Maximal ATP yield would be achieved if the entire free energy difference could be conserved as ATP. However, in that case the reaction would be in thermodynamic equilibrium, and thus rates of substrate degradation and ATP production would be zero. Part of the free energy difference must to be used to drive the reaction. The larger this part is, the faster the rate of ATP production but also the lower the yield. High yield is a group-beneficial trait because the economic utilization of a resource benefits all those sharing this (limiting) resource. On the other hand, high growth rate is beneficial for the individual because it allows better competition. Cooperative behavior, resulting in higher yield, was found to outweigh the interest of the individual to grow faster in spatially structured environments, such as biofilms (Pfeiffer et al., 2001).

Evolutionary trade-offs have also impacted metabolic network design, and its regulation. Species inhabiting complex, dynamic environments have metabolic networks in which enzymes have relatively more connections to other enzymes than species living in more constant environments. This makes their networks more robust, but also more costly to maintain and thus less efficient (Morine et al., 2009). Under nutrient scarcity, cheap but less efficient pathways are expressed (Carlson, 2007), in which the length and elemental content of proteins may be adapted to the nutrient limitation (Elser et al., 2011). These metabolic network characteristics appear identifiable from the genome sequence and as such may be incorporated in future multispecies FBA approaches.

The above optimization criteria put emphasis on the fitness of the individual cell and species, as goal in ecosystem development. A framework of ecological network analysis, employing community-level, thermodynamics-motivated flux optimization criteria (Kleidon et al., 2010) has been postulated as alternative theory to describe the goal of ecosystems (Fath, 2004; Ulanowicz et al., 2006; Jørgensen, 2007), and has so far mainly been applied to plant and animal ecology, e.g., to predict species distributions (Phillips et al., 2006). Maximum entropy principles have also allowed for predicting species abundances within plant communities (Shipley et al., 2006), although some of its assumptions have been criticized too (e.g., Laughlin et al., 2012). Recently, the utility of multi-level optimization was revealed for purely metabolic models of microbial consortia (Zomorrodi and Maranas, 2012). An optimization approach was formulated with maximization of the overall biomass as primary, community level-objective function and species-specific biomass maximization as secondary, cellular objective function. This approach allowed for capturing any type of positive or negative interaction, like mutualism, competition and parasitism, and demonstrated the trade-offs between forces driving species and community fitness. It would be interesting to further combine community-level and cell-based optimization approaches in microbial ecology, and oil spill biodegradation in particular, to establish what level of integration is required to describe microbial community functioning.

## Deriving microbial physiological parameters for application in FBA

It is essential to be aware that besides information on metabolic pathways and optimization criteria, FBA models also require basic physiological data: a growth-associated maintenance (energy expenditure necessary for non-metabolic activities accompanying biomass synthesis, usually expressed in mmol ATP per gram biomass per hour), growth-rate independent maintenance (or non-growth associated maintenance, the energy needed to maintain the cell in a functional and viable state, without growing), efficiency of respiration [P/e; amount of ATP (P) produced from the movement of an electron (e) through an electron transport chain to an electron acceptor] and biomass composition. Some of these variables might be of less importance in a FBA context than generally conceived. For instance, determining the composition of biomass is tedious, in particular for species within a community, and is highly responsive to changing environmental conditions (e.g., Pramanik and Keasling, 1997, 1998). However, when biomass composition was varied in an *E. coli* FBA model, it had only a minor influence of its modeled growth rate and oxygen uptake rate (Feist et al., 2007). This suggests that biomass composition can be neglected, although it will affect intracellular fluxes (Pramanik and Keasling, 1997, 1998) and trophic interactions (Matz and Kjelleberg, 2005). Similarly, growth-rate independent maintenance energy requirements are tedious to determine and interpret (Van Verseveld et al., 1984). However, *in situ* maintenance energy is much lower than the maintenance energies determined in the laboratory (Morita, 1988), suggesting that growth-rate independent maintenance energy is also not required for modeling, while it may also be estimated from thermodynamic approaches (Tijhuis et al., 1993). The inclusion of growth-associated maintenance, on the other hand, is essential, as microbial metabolism is inefficient: the amount of biomass produced per mole ATP is much less than theoretically possible (Stouthamer, 1973). Westerhoff et al. (1983) described that low thermodynamic efficiencies are optimal for maximum growth, and established a relation between the reduction grade of substrate and efficiency.

FBA models predict growth yields. Given that thermodynamic models more directly predict yield estimates (Vanbriesen, 2002; Roden and Jin, 2011), these estimates may utilized as additional constraints in FBA. A literature compilation demonstrated a linear relationship between measured microbial growth yield and the free energy of aerobic and anaerobic respiratory and fermentative metabolism of glucose, organic acids, ethanol, and hydrogen (Roden and Jin, 2011). An initial prediction of growth yield on basis of thermodynamics may in particular aid fitting P/e ratios from FBA models for respiring microorganisms. Oxidative phosphorylation is generally the major source for ATP in respiring microorganisms, including those active in oil spill degradation, but it is experimentally challenging to quantify its P/e.

**Figure 4:**
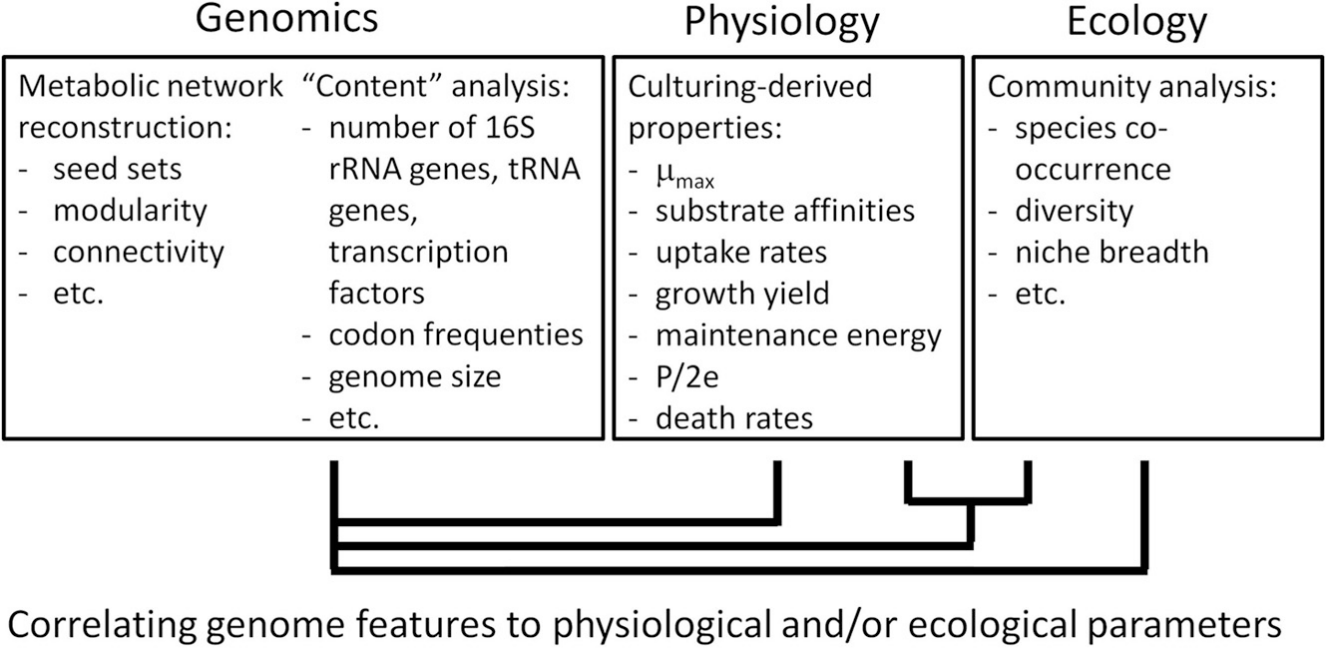
**From genome to physiology and ecology of a species**. The interaction with their environment over evolutionary times has shaped the physiology of microorganisms, which is contained in their genomes. We assume that, in reverse, the genome informs on a species' physiology and ecology. This figure indicates how one may derive relationships between genomic information on the one hand and physiological and ecological characteristics on the other hand. These relationships or “rules” will further aid the modeling of microbial communities and functioning by bottom-up approaches.

Most of the above mentioned physiological parameters would also be needed for other modeling approaches, e.g., kinetic models. Determining these parameters is time-consuming and poses a serious constraint on modeling complex microbial communities in detail, also calling for a more optimal use of the large amount of physiological data collected in the past (see Section “Concluding Remarks”). Paradoxically, top–down approaches (Figures 2, 4) may also aid in achieving bottom up-modeling of complex communities: empirical relations may be used to infer physiological parameters or first model activities, and can later on be replaced by more mechanistic descriptions when more insight is obtained. For instance, genome composition and genome-derived metabolic network structure of a microorganism inform on physiological characteristics such as growth rates (Freilich et al., 2009, 2010; Sharp et al., 2010; Vieira-Silva and Rocha, 2010), since they are shaped by the environment in which the microorganism evolved. rRNA operon copy number, tRNA copy number and a composite index of codon usage bias derived from (meta)genome sequence data correlate with maximum growth rate (Sharp et al., 2010; Vieira-Silva and Rocha, 2010). Metabolic variability and co-habitation (or competition) encountered can be derived from genome sequence data, and also correlate with growth rates (Freilich et al., 2009). Such rates, together with principles of biochemistry and genomics (e.g., Beg et al., 2007; Henry et al., 2007; Chandrasekaran and Price, 2010), may be used to constrain FBA models. A quantitative description of substrate uptake rates in multispecies FBA is in particular important for modeling competition and also requires information on substrate affinities. Recently, evidence was obtained that kinetic parameters, such as affinity constants, correlated with the amino acid composition of enzymes (Zikmanis and Kampenusa, 2012).

## Concluding remarks

The road toward understanding and predicting microbial community functioning is clearly still long and will be challenging. Achieving this goal will require a more optimal, integrative use of the enormous amount of data that is already available. Current microbial ecology is strongly dominated by molecular analyses, however the “old” microbial physiology data are still of high value to give further meaning to molecular-based community analysis. Yet, the data are spread over a large number of publications. In biochemistry, kinetic parameters for enzymes, such as turn-over rates, affinity constants, maximum rates have been compiled in the BRENDA database (Schomburg et al., 2013). We advocate establishing a similar database with kinetic parameters of microorganisms, and also containing information on their physiology and ecological characteristics.

Such a database will help to advance microbial ecology in two ways. Firstly, phylogenetic information, as derived from 16S rRNA sequencing or metagenomics, might be linked to the closest relative in such a database to automatically extract its kinetic properties and ease modeling of the ecosystem from which the community data were derived. Secondly, a database with microbial physiology data will facilitate the large-scale correlation of physiological data with genome information (Figure 4). This enables deriving “rules” on microbial physiology and ecology (Borenstein et al., 2008; Freilich et al., 2009, 2010), which may aid bottom-up approaches, e.g., to derive the mechanistic reasons behind observed patterns (Figure 2).

In addition, more microbial physiological data will be needed, particularly on the impacts of co-cultivation on the physiology of individual species and their interactions. In addition, while microorganisms of industrial and clinical relevance were well-characterized in monocultures in the 1970s to 1990s, advanced culturing methods have now allowed for isolation of species from phyla which were not even described at that time. Comparative microbial physiology studies, on novel single species or mixed species cultures, will benefit modeling.

We have provided a synthesis on how approaches currently available can be integrated and extended to computationally derive multispecies, metabolic flux networks from metagenomic data. This integration will contribute to answering key questions in microbial ecology, such as understanding the assembly of microbial communities and their functioning via metabolic interactions. Oil-degrading communities are often simple and dominated by culturable species, providing a suitable, tractable test system. In recent years, an enormous amount of largely descriptive studies on the degradation of marine oil spills has appeared (e.g., Camilli et al., 2010; Hazen et al., 2010; Lu et al., 2012; Mason et al., 2012). This wealth of data can be further analyzed to establish the co-occurrence and dynamic interactions between key microorganisms contributing to oil-degradation. The available genomic information on key isolates (Schneiker et al., 2006) and on non-culturable key players through single cell sequencing (Mason et al., 2012) can be included in the multi-species metabolic models. This modeling, in iteration with well-designed experiments, should substantially enhance our knowledge on the biodegradation of marine oil spills.

### Conflict of interest statement

The authors declare that the research was conducted in the absence of any commercial or financial relationships that could be construed as a potential conflict of interest.
